# A Case of Acute Human Self-Poisoning With Bispyribac Sodium Presenting as Lactic Acidosis

**DOI:** 10.7759/cureus.65454

**Published:** 2024-07-26

**Authors:** Sreejith Jayachandran, Nidhi Kaeley, Parvathy Sasidharan, Joen R Mathew, Amrita Paul

**Affiliations:** 1 Emergency Medicine, All India Institute of Medical Sciences, Rishikesh, Rishikesh, IND; 2 Family and Community Medicine, All India Institute of Medical Sciences, Rishikesh, Rishikesh, IND

**Keywords:** agricultural poisoning, toxicology, lactic acidosis, herbicide poising, bispyribac sodium

## Abstract

This case report describes a 65-year-old male who presented to the emergency department with significant lactic acidosis after self-poisoning by ingesting bispyribac sodium, a commonly known herbicide. This case highlights the rarity of poisoning with freely available herbicides in the literature, which may be elusive in clinical history and life-threatening in presentation. The patient had attempted to commit suicide with ingestion of an unidentified herbicide and was brought to the emergency department post two hours after the incident. He complained of abdominal pain. The hemodynamics of the patient were within normal limits. However, his initial lactate levels were elevated along with a high anion gap metabolic acidosis. The patient was provided symptomatic care and close monitoring. The ingested substance was later found to be bispyribac sodium. The patient symptomatically improved over time, with lactate levels attaining normal ranges, and was discharged after observation of 24 hours. Human ingestion of bispyribac sodium is mostly asymptomatic and non-fatal. The management in this case mainly consisted of symptomatic care. The initial presentation of herbicide poisoning in an emergency department setting as lactic acidosis and the subsequent evaluation to rule out other possible causes of lactic acidosis in the patient was challenging for the treating physician. The possibility of herbicide-mediated cellular damage and subsequent lactic acidosis is thought to be the reason for this rare presentation.

## Introduction

India relies heavily on agricultural output, which makes up a majority of its GDP. As a result, it ranks 12th in the usage of pesticides globally and is ranked third in Asia after China and Turkey [1]. Apart from agricultural chemicals posing a threat of environmental contamination, deliberate self-ingestion or poisoning by such chemicals has been on the rise in the last few years. India recorded a suicide rate of 14.04 per 100,000 population in 2019 and is ranked 49th globally [2]. It is estimated that 15,000 farmers commit suicide in India every year, out of which more than 50% is from ingestion of highly toxic pesticides. Moreover, there is a reported mortality of 60,000-70,000 each year due to pesticide ingestion by children and adults not related to agriculture who live in rural communities [3]. Pesticides may be classified as containing herbicides, insecticides, or fungicides. Indian literature has scarce data regarding poisoning cases with herbicides, even though they are widely available. Most of the cases of herbicide poisoning are underreported, likely due to the inability to differentiate them from cholinergic pesticides. Due to the non-availability of specific clinical symptoms, the diagnosis is mainly based on clinical history.

Lactate level is a useful clinical parameter often used in emergencies, with high levels associated with increased mortality rates in patients suffering from sepsis. Lactate clearance has been considered as a prognostic marker in sepsis patients [4]. Herbicide poisoning in humans presenting as lactic acidosis has been reported previously, with less clarity on its pathophysiology. Here, we present a case of acute self-poisoning in a patient with bispyribac sodium, a commonly used herbicide, presenting to the emergency department with the complaint of lactic acidosis; a complete diagnosis was made only after the complete recovery of the patient.

## Case presentation

The patient is a 65-year-old male who presented to the emergency department (ED) after claiming to have consumed 50 mL of an unknown substance. Following ingestion, the patient developed epigastric pain and had a few episodes of vomiting. The patient denied any symptoms such as constipation, diarrhea, dysuria, decreased urinary output, chest pain, or shortness of breath. He described his epigastric pain as non-radiating, which had developed after ingesting the unknown substance two hours prior to presentation. The patient was brought into the ED two hours post ingestion. Upon arrival at the emergency department, the patient's airway was confirmed to be patent. His vitals showed a heart rate of 82/min, blood pressure of 120/80 mmHg, respiratory rate of 16/min, peripheral oxygen saturation was 96% in room air, and auscultation showed normal vesicular breath sounds bilaterally in all lung fields. The patient had a Glasgow Coma Scale (GCS) score of E4V5M6. His pupils were 3 mm and reactive bilaterally. The body temperature was measured to be 97.6 degrees Fahrenheit and there was no suspected characteristic smell of poisoning. The point-of-care blood sugar value was within normal ranges. An ECG showed normal sinus rhythms, with no specific ST- or T-segment changes. His laboratory results are provided in Table 1. 

**Table 1:** Laboratory results of the patient with corresponding reference ranges. Hb: hemoglobin; SGOT: serum glutamic-oxaloacetic transaminase; SGPT: serum glutamic pyruvic transaminase; g/dl: grams per decilitre; cu mm: cubic millimeter; mg/dL: milligrams per deciliter; mmo/L: millimoles per liter

Laboratory Parameters	Observation Results	Reference Ranges
Hb%	12.9 g/dL	13-17 g/dL
Total leucocyte count (TLC)	13,600/ uL	4000-11000/ uL
Platelet count	220,000 cells/cu mm	150,000-400,000 cells/cu mm
Serum creatinine	1.9 mg/dL	0.55-1.02 mg/dL
Blood urea	33 mg/dL	17-43 mg/dL
Serum sodium	140 mmol/L	136-146 mmol/L
Potassium	4.6 mmol/L	3.5-5.1 mmol/L
Chloride	102 mmol/L	101-109 mmol/L
Total bilirubin	0.9 mg/dL	0.3-1.2 mg/dL
SGOT/SGPT	24/37 U/L	0-35 U/L
Serum albumin	4.3 g/dL	3.5-5.2 g/dL

The initial lactate value was 9.2 mmol/L. The patient was managed conservatively. Symptomatic care included management of abdominal pain with intravenous paracetamol (1 gram IV) which was continued in an oral form. Intravenous fluids were also administered. Adequate fluid status was maintained for the patient. Further arterial blood gas sampling showed a decreasing trend of lactate levels. The patient's symptoms resolved within a few hours and the patient remained asymptomatic during the further observation period. Later, it was found out from the patient's attenders that the ingested substance was bispyribac sodium (sold under the brand name Dry-Up Gold in India). His lactate levels normalized in 12 hours. Following observation in the emergency department, the patient was later discharged with full resolution of symptoms and normal lactate levels.

**Figure 1:**
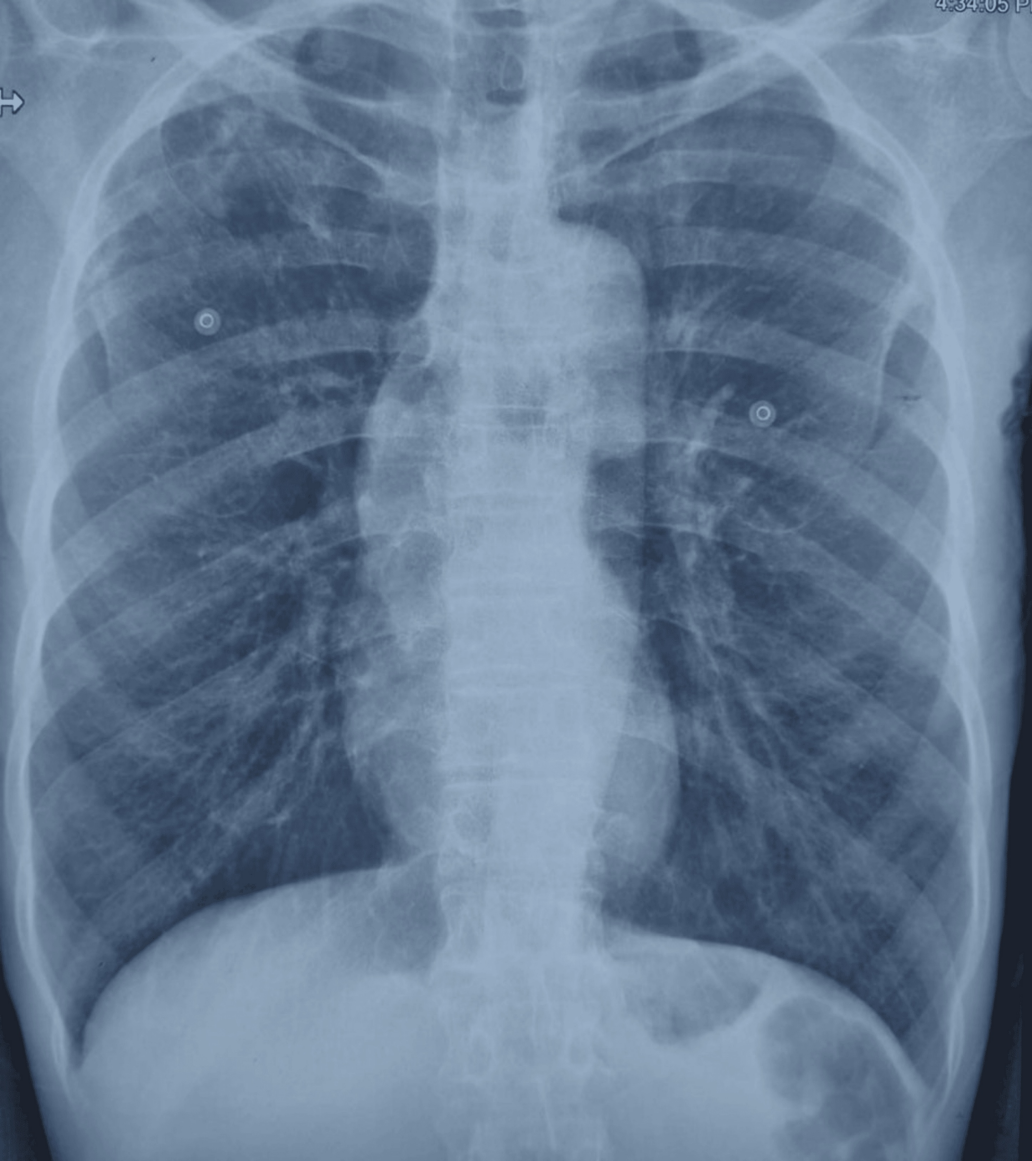
Anterior-posterior view of chest X-ray of the patient with normal bony structures, airway passages, lung parenchyma, and cardiac shadow.

**Figure 2:**
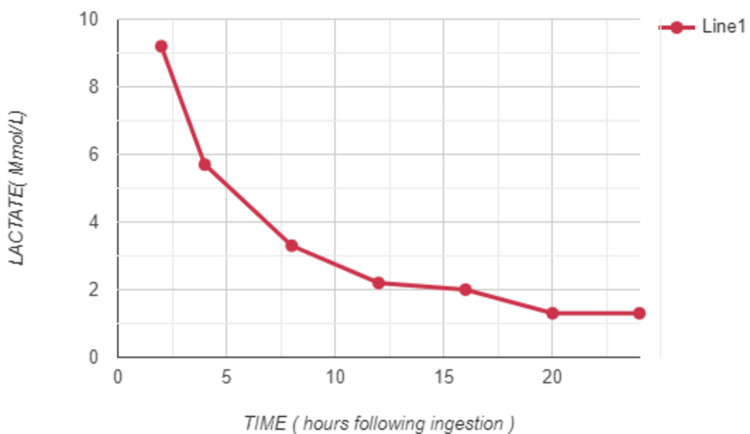
The graph shows the lactate levels (in mmol/L) of the patient obtained from serial arterial blood gas analysis showing decreasing levels of lactate over time (in hours).

## Discussion

Bispyribac (KIH-2023, figure 1) inhibits the enzyme acetolactate synthase. It is used as a systemic post-emergence herbicide, which is used to control the growth of weeds in agriculture [5]. Even though it is a common herbicide used in India, the clinical literature shows only a few reported cases of acute ingestion with bispyribac sodium. Due to its clinical presentation consisting of non-specific symptoms, it poses a diagnostic difficulty for clinicians. With symptoms being related mostly to altered mental sensorium with a low GCS score of 6, the suspicion of poisoning with bispyribac sodium is rarely raised in the emergency department. In a prospective observational study, the clinical outcomes of patients who experienced bispyribac poisoning appear to favor other commercially available herbicides with a case fatality ratio calculated to be less than 5.8% [6]. Common cases of acute human poisoning that are known to present with lactic acidosis are salicylate, ethylene glycol, methanol, and propylene glycol [7].

Lactic acidosis results in high anion gap metabolic acidosis. Lactic acidosis is mainly of two types. Type A lactic acidosis which is due to tissue hypo-perfusion and high levels can be fatal causing accelerated deterioration of clinical condition. Type B lactic acidosis is not related to tissue ischemia and is therefore less fatal. Type 2 lactic acidosis is seen in seizure patients, alcoholics, and in the usage of drugs like metformin. A case report on metolachlor self-poisoning [8], a commonly used herbicide, had a presentation of lactic acidosis due to the formation of methemoglobin, which caused hypoxia and lactic acidosis. Another case report on glyphosate self-poisoning [9], a herbicide causing toxicity via uncoupling of oxidative phosphorylation, also reported elevated levels of lactate.

The management of acute human poisoning with bispyribac sodium is mainly supportive care and reassurance. The airway has to be maintained in case of patients presenting with low GCS. Adequate volume status has to be maintained with frequent hemodynamic monitoring. Because of its comparatively low toxicity, gastric decontamination especially lavage or forced emesis is discouraged [10]. This case report suggests that acute human poisoning with bispyribac sodium can involve the presentation of lactic acidosis. Despite management being mainly conservative, care should be given to the hemodynamic instability that can arise due to elevated lactate levels [11].

**Figure 3:**
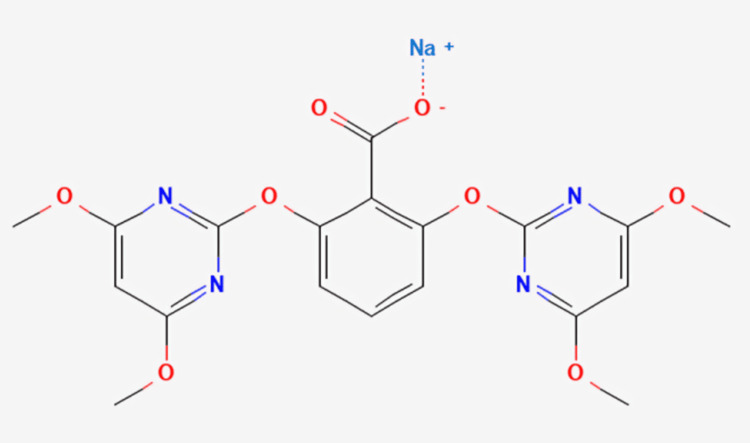
Chemical structure depiction of bispyribac sodium National Center for Biotechnology Information (2024). PubChem Compound Summary for CID 23682789, Bispyribac-sodium. Retrieved July 7, 2024 from https://pubchem.ncbi.nlm.nih.gov/compound/Bispyribac-sodium [12].

## Conclusions

Human ingestion of bispyribac sodium is mostly asymptomatic and non-fatal. Here we report a case of bispyribac sodium poisoning which presented at the emergency department as type 2 lactic acidosis with abdominal symptoms. The management mainly consisted of symptomatic care, with most of the symptoms resolving with conservative management. The elevated lactate levels are likely due to toxin-mediated cellular metabolism, with no hemodynamic compromise occurring during the course of treatment and observation. 
